# Three-dimensional quantitative analysis of temporal region morphology in Chinese young adult

**DOI:** 10.7717/peerj.14226

**Published:** 2023-02-02

**Authors:** Yumeng Wu, Chongmai Zeng, Duanyu Feng, Zhilong Chen, Qian Fu, Wen Liao

**Affiliations:** 1 State Key Laboratory of Oral Diseases, National Clinical Research Centre for Oral Diseases, West China Hospital of Stomatology, Sichuan University, Chengdu, Sichuan, China; 2 School of Mathematics, Sichuan University, Sichuan University, Chengdu, Sichuan, China; 3 State Key Laboratory of Oral Diseases & National Clinical Research Center for Oral Diseases, Department of Orthodontics, West China Hospital of Stomatology, Sichuan University, Sichuan University, Chengdu, Sichuan, China

**Keywords:** Temporal region, Morphology, 3dMD, Soft tissue, Quantitative analysis

## Abstract

**Background:**

Temporal filling is commonly used to correct temporal depression. However, there is a lack of quantitative criteria for pre- and post-operative evaluations. The use of three-dimensional scanning may help improving the success of temporal filling by providing quantitative assessments. The study aimed to compare the results of qualitative morphological evaluation of the temporal region with a quantitative, numerical analysis of the temporal difference value (TDV).

**Methods:**

We enrolled twenty-six male and forty-nine female volunteers aged 18 to 29 years. Facial images were acquired in OBJ format using 3dMD facial stereo-photography. The morphologies of the temporal regions were separately evaluated by four researchers in the form of two-dimensional (2D) images. Results were classified as either aesthetic or unaesthetic. The quantitative evaluation of the temporal region was then conducted. First, the temporal region was trimmed out from the original 3D image into a new OBJ file. Second, interpolation was used to construct a smooth, adapted surface. Third, a mathematical model of temporal region flatness denoted as the TDV, which was defined as the sum of the Euclidean distances of all 3D points between the constructed surface and the temporal-region OBJ file. The classification of each sample was compared with its TDV to verify the mathematical model’s validity. The cutoff threshold and prediction accuracy of this mathematical model were calculated.

**Results:**

The cutoff threshold between aesthetic and unaesthetic TDV was found to be 24.66 for males and 28.11 for females. The prediction accuracy rate was 0.73 for men and 0.73 for women.

**Conclusion:**

The method has high overlap and good repeatability and minimizes the influence of subjective aesthetics on morphological judgment. TDV has a certain reference value for clinical temporal region evaluation.

## Introduction

Temporal regions are areas of the upper-mid-face in which the contours of the face are relatively prominent. Facial adipose loss is a major contributor to facial aging, and a fuller face often left with a younger appearance and impression (Shaw & Kahn, 2007). Different facial areas undergo adipose compartments deflation at different rates. Atrophy of these adipose tissues causes uneven transition points between different facial areas. Such a process will create defects such as deep folds, wrinkles, and a hollowed appearance, which are considered aging characteristics (Rohrich & Pessa, 2007). Therefore, depressions in these areas create deep shadows, make the signs of aging more noticeable and affect the combined face (Tzikas, 2018). Younger Asian patients tend to ask for facial rejuvenation, focusing on improving facial shape and its three-dimensional form, which reflects their desire to improve the underlying structure to serve their ideal facial aesthetics (Liew et al., 2016).

Grading evaluations, such as photonumeric scale evaluation, are 2D evaluation methods often performed pre- and postoperatively for temporal surgery as they provide quick clinical data and can widely be used in clinical practice (Weissler et al., 2017). However, such evaluations are subjective; hence, results could be influenced by patients’ skin color, light, and shadow (Plooij et al., 2009), which may lead to overfilling or insufficient filling. Some physicians may only make qualitative diagnoses during the preoperative phase and utilize intraoperative observations during depression removal as an indicator of the completion of surgery (Giacomo et al., 2021). Thus, there is an urgent need for an objective, unified, and easy-to-use criteria for the pre-and postoperative evaluation of temporal-region morphology.

Photonumeric scale evaluation has been used to evaluate facial appearance for a long time. In recent years, the use of three-dimensional (3D) stereoscopic photography technology has emerged in medical fields. The non-invasive nature and precision of this technique make it a valuable modality in the fields of plastic surgery, craniofacial surgery, and orthodontics (Liu, Xu & Lin, 2008; Choi et al., 2014; Maal et al., 2010); treatment changes, surgical simulations, and many other surgical applications were then reachable. The 3dMD System (Atlanta, GA, USA) is based on 3D stereoscopic photography and produces an image of the whole face within 1.5 ms through an active stereo approach which eliminates ambient spectral interference (Incrapera et al., 2010; Schaaf et al., 2010; Weinberg et al., 2006). The anti-interference capabilities and fast stereo-reconstruction speed of the system make it ideal for facial plastic surgery, as clinicians can obtain facial features’ 3D spatial information, which is difficult to acquire using 2D measurements or visual inspection alone (Dindaroğlu et al., 2016).

2D scoring is currently the most widely used to measure facial morphology (Weissler et al., 2017), which is broadly applicable but, to some extent, influenced by subjective differences in the evaluator. 3D scanning can yield relative accurate data, but such objective morphological data does not include subjective aesthetic evaluation.

Quantitative analyses bridge the gap between subjective aesthetic evaluations and objective measurement data. Such analyses can provide beauty seekers and professionals with more objective treatment suggestions because 3D images can provide more specific information (Kovacs et al., 2006). The present study aimed to compare the qualitative morphological 2D evaluation’s result with a quantitative, numerical 3D analysis of the TDV. We also explored the possibility of using mathematical methods to evaluate soft-tissue aesthetics, aiming to provide a possible theoretical basis for rapid aesthetic evaluation.

## Materials and Methods

### Inclusion criteria

The study samples consist of 75 subjects, including 26 males and 49 females. Inclusion criteria were: (1) aged 18–29 years old Chinese and (2) No discerning facial deformity, in good general health with relatively a symmetrical face, no history of facial surgery, and no obvious scars, birthmarks or any skin diseases in the temporal region. All volunteers provided informed written consent for using facial photos in analyses and publication. This study was approved by the Ethics Committee of West China Hospital of Stomatology, NO. WCHSIRB-D-2020-256.

### Two-dimensional photography

**Figure 1:**
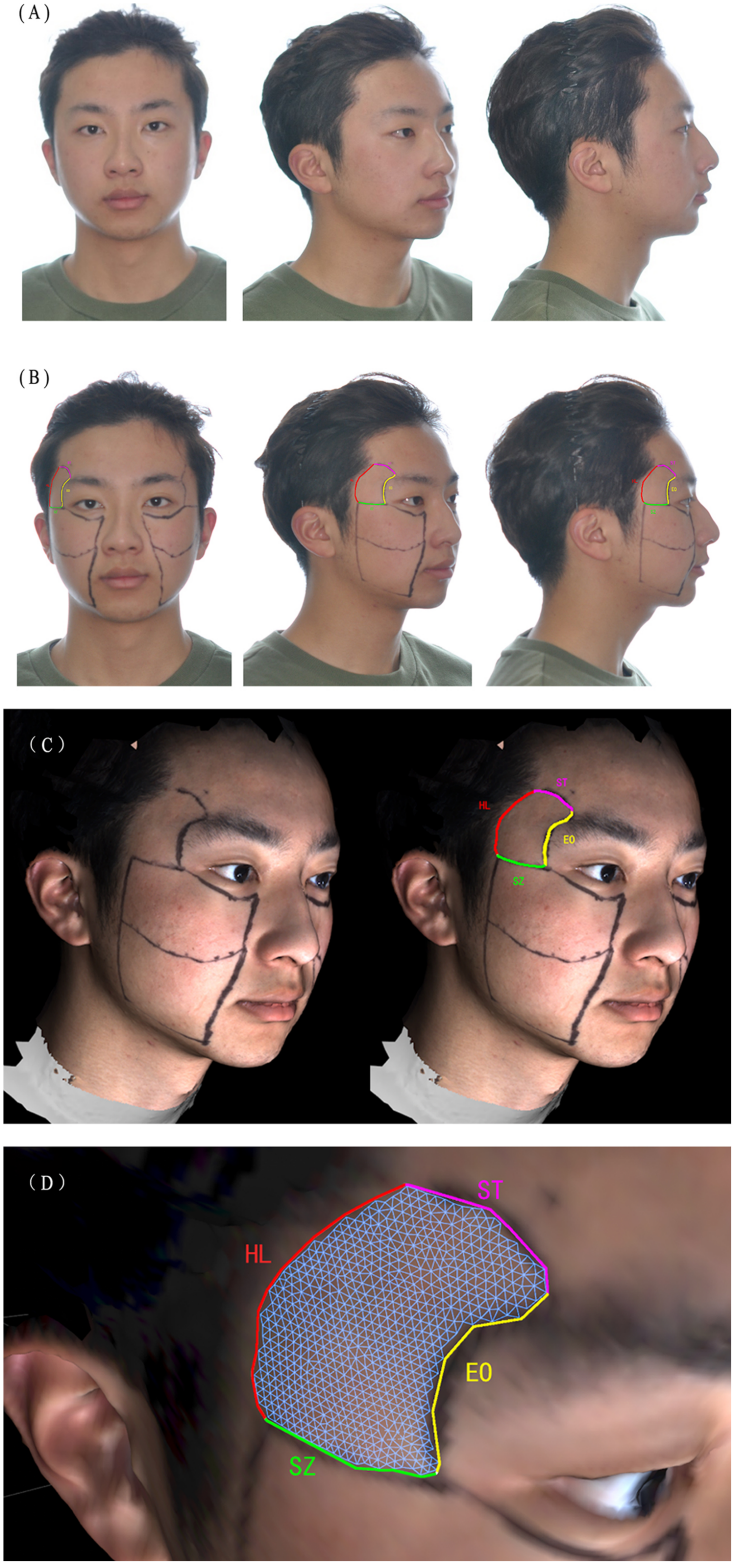
The acquiring of 2D images and trimming of temporal regions. (A) The individuals were instructed to maintain a natural head position. (B) Individual wearing facial marker lines which assisted in positioning temporal regions. The soft-tissue temporal region was defined by the superior temporal line as the superior boundary (ST), the hairline as the posterior boundary (HL), the superior border of the zygomatic arch as the inferior boundary (SZ), and the external orbit rim as the anterior boundary (EO). (C) The facial marker lines on 3D model. (D) Close look of temporal region. The temporal region is trimmed.

Two-dimensional (2D) facial images were acquired using a digital camera (N90; Nikon Inc., Tokyo, Japan). Individuals were instructed to maintain a natural head position while 2D images were taken from five angles (front, 45°, and 90° on both sides) with two expressions (smiling and natural facial expression) (Fig. 1A).

### Placement of fixing points

Facial marker lines were placed on the participants to identify fixed points for the 3D models. Such a method was developed to conquer the challenges of temporal soft tissue margin identification after image scanning. The superior temporal line defined the soft-tissue temporal region as the superior boundary, the hairline as the posterior boundary, with the superior border of the zygomatic arch being the inferior boundary, and the external orbit rim as the anterior boundary (Carruthers et al., 2016) (Fig. 1B).

### Generation of three-dimensional models

After obtaining participants’ 3D facial images with the 3dMD facial stereo-photography System (Fig. 1C), the system software was used to generate 3D facial image data (OBJ format). All 3D facial photographs were taken according to the manufacturer’s operation protocol.

### Trimming of the three-dimensional models

The 3D facial images were processed in OBJ format using Cinema 4D (R16), trimming along the marker lines as shown in Fig. 1D and dividing by cutting along the marker lines. Four researchers independently extracted the temporal regions of both sides, and data were exported in OBJ format.

### Rating of two-dimensional photography and three-dimensional models

Each researcher rated the natural-expression 2D photographs of each angle four times at 2-week intervals to avoid subjective factors as much as possible. To this end, aesthetic evaluations of the temporal region were carried out, and each image was rated as mildly concave (+), flat (0), or mildly convex (−) according to the Allergan Temple Hollowing Scale (Carruthers et al., 2016). Finally, we classified mild concavity (+) and mild convexity (−) as unaesthetic and flat as aesthetic. The interclass correlation coefficient (ICC) was calculated using SPSS (Version 22.0; IBM, Armonk, NY, USA). The ratio of the rating was used to calculate the accuracy rate. Since the rating process is considered subjective, the accuracy rate was then calculated several times to get the final ratings provided in Table 1.

**Table 1:** The rating of 2D images performed by four researchers. The research ran through a collation of the accuracy rate of the rating.

	Researcher 1	Researcher 2	Researcher 3	Researcher 4
First rating	0.81	0.93	0.61	0.57
Second rating	0.91	0.96	0.89	0.57
Third rating	0.91	0.96	0.89	0.80
Fourth rating	0.97	1.00	0.95	0.85

### Mathematical analyses

**Figure 2:**
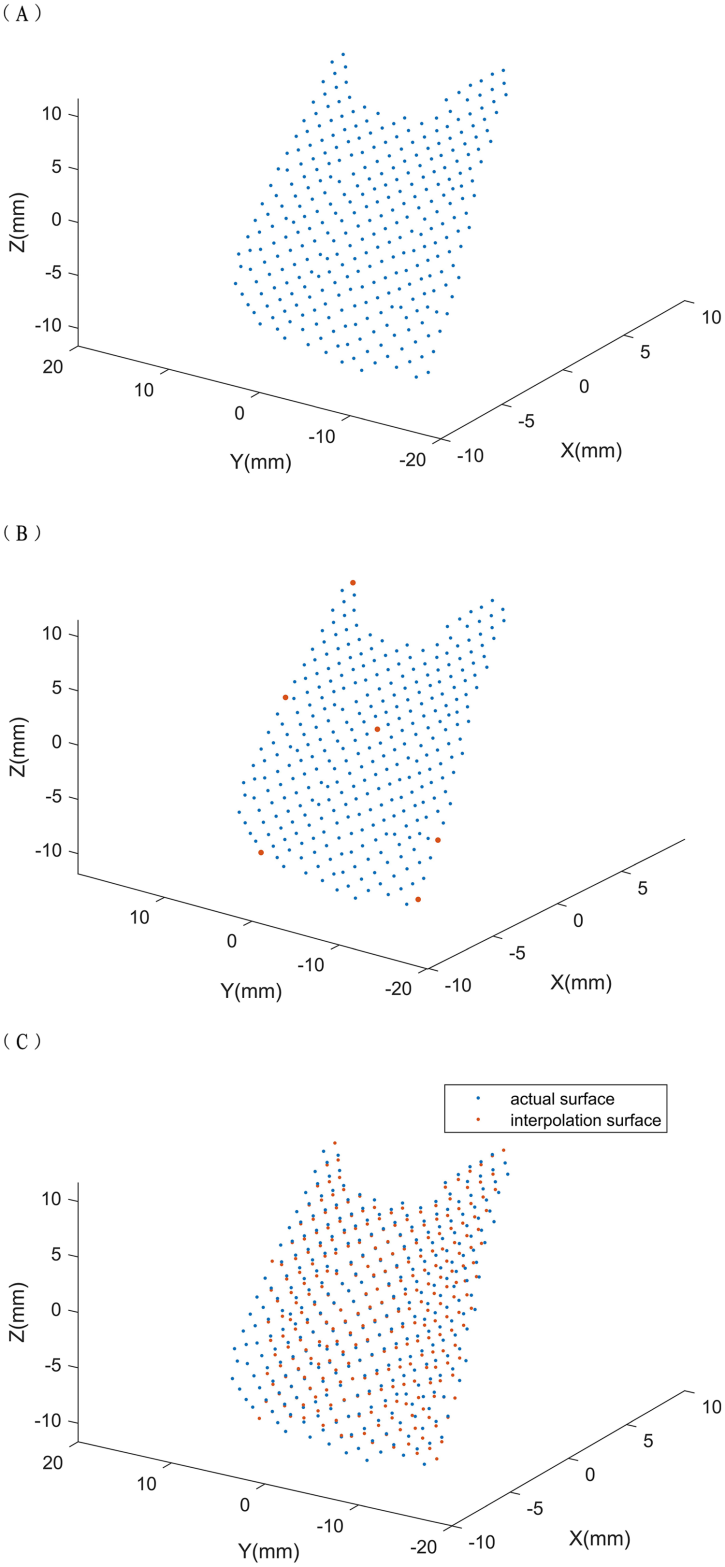
(A-C) Temporal region’s soft tissue composed of a three-dimensional point cloud. The three-dimensional point cloud represents temporal region’s soft tissue, the characteristic points are taken and the interpolation surface is calculated. We selected the following points (red points) as interpolation points: the maximum and minimum points on the x, y, and z axes of the 3D point cloud and the point closest to the center of gravity (x1, y1, z1) of the point cloud, which is generally the center point of the 3D point cloud.

Importing the OBJ data of the trimmed soft tissue of the temporal regions into MATLAB (R2016b; MathWorks, Inc., Natick, MA, USA) resulted in the generation of surface data of a 3D point cloud $\left( {\rm x,y,z} \right)$, which represented the temporal-region soft tissue (Fig. 2A). We built a model and extracted information relating to convexity and smoothness of the surface, and integrated 3D point clouds to automatically determine the temporal region soft tissue is aesthetic/unaesthetic.

We took some characteristic points of the surface and conducted 3D interpolation to construct a new surface. We then used triangle-based cubic interpolation so that the first derivative was continuous (Strang & Fix, 1975) to ensure the smoothness of the generated surface. Because the aesthetic surface should exhibit continuity, the degree of convexity and smoothness of the interpolated surface should be close to aesthetic. Therefore, comparing the new and actual surfaces enabled the soft tissue of the temporal region to be identified as aesthetic or unaesthetic.

We selected the following points as interpolation points: the maximum and minimum points on the x, y, and z axes of the 3D point cloud and the point closest to the center of gravity (x_1_, y_1_, z_1_) of the point cloud, which is generally the center point of the 3D point cloud. We used the Euclidean distance (d) to judge which point is the closest. Here, d is mathematically expressed as:

$d = \sqrt {{{\left( {x - {x_1}} \right)}^2} + {{\left( {y - {y_1}} \right)}^2} + {{\left( {z - {z_1}} \right)}^2}}$

The point selection is illustrated in Fig. 2B.

The points were used for triangle-based cubic interpolation, to obtain a new interpolated surface (Fig. 2C), and to calculate the sum of all 3D Euclidean distances between the interpolated surface and actual surfaces. The sum was divided by the number of points to provide the TDV. Later, we will judge the state of the temporal region’s soft tissue based on this value and other factors.

### Statistical analyses

Logistic regression model is commonly used in medicine to evaluate the risk of illness (Hart et al., 2020; Xing et al., 2019), as well as to test the relationship between specific parameters and disease risk (Zhou et al., 2019). Therefore, we used a logistic regression model to analyze the relationship between specific parameters and the morphology of the temporal region. Prior to logistic regression analysis, the data were processed as follows:

Four operators independently trimmed the soft tissue of each temporal-region image to generate 3D point clouds. To minimize intra-operator error, each sample was trimmed by four operators in 3D, and a mixed model was used to synthesize four TDVs and then obtain an average TDV.

An accurate assessment of the influence of variables such as gender (g), age (a), and body mass index (BMI) (b) was carried out by inputting the state of the temporal-region soft tissue as the dependent variable and using gender, age, BMI, and average TDVs as independent variables for the logistic model.

After adjusting the probability cutoff threshold, we compared the accuracy rates obtained by cross-validation for each threshold. Finally, we selected a probability cutoff threshold of 0.55 and used this value to reversely solve the cutoff threshold for TDV using average age and BMI values for males and females instead of actual data. Therefore, when new data are collected, we can assess the state of aesthetics by simply trimming and calculating the TDV then comparing with the cutoff threshold of the TDV.

## Results

We enrolled 75 participants, 26 males and 49 females, with an average age of 20.18 and an average BMI of 20.60. We calculated sample size by setting the alpha, power, delta, and the estimated sensitivity as 0.05, 0.80, 0.2 and 0.7, respectively. The estimated sample size was therefore 49. The ICC value of the rating was 0.711. The cutoff threshold of the TDV was 0.045 for males and 0.084 for females. The prediction accuracy rate of the model was found to be 0.73 for men and 0.73 for women.

The causes of rating errors in the model are shown in Table 2A.

**Table 2:** The accuracy rate of the rating method of the mathematical model & the accuracy rate of interpolation model. (A) To analyze the causes of the rating errors in the model that the research has constructed, the experiment needs to count the specific conditions based on the accuracy rate of our mathematical rating method. (B) Based on the analysis, to minimize the misjudgment that occurs when the samples of males were normal and the samples of females were abnormal, the experiment attempted to set the lower bound of the 95% confidence interval of the abnormal average TDV as the cut-off value under the gender classification and get Table 2B.

(A) The accuracy rate of the rating method of the mathematical model					
		Less than the cutoff value (normal)	Greater than the cutoff value (abnormal)	Total	Prediction accuracy
Male	Normal	3	6	9	0.33
	Abnormal	1	16	17	0.94
	Total	4	22	26	0.73
Female	Normal	35	1	36	0.97
	Abnormal	12	1	13	0.08
	Total	47	2	49	0.73
**(B) The accuracy rate of interpolation model**					
		Less than the cutoff value (normal)	Greater than the cutoff value (abnormal)	Total	Prediction accuracy
Male	Normal	5	4	9	0.56
	Abnormal	4	13	17	0.76
	Total	9	17	26	0.69
Female	Normal	11	25	36	0.31
	Abnormal	2	11	13	0.85
	Total	13	36	49	0.45

Based on the cutoff value, the data indicates that misjudgment is likely when the temporal region is rated as aesthetic, which could be due to low number of aesthetic samples included in the data set. Therefore, the logistic regression model would have rated more subjects as aesthetic after training.

The average TDV, obtained by counting through our interpolation model and further analyzing the model’s separation of aesthetic and unaesthetic, is shown in Table 3.

**Table 3:** Statistics of average TDV. The average TDV was obtained by counting through our interpolation model and further analyzing the interpolation model’s separation effect on normal and abnormal.

	Mean	Median	Standard deviation	Interquartile range	95% confidence interval
Male (normal)	0.053965	0.048668	0.012888	0.023876	[0.044059–0.063872]
Male (abnormal)	0.061315	0.057929	0.009926	0.015039	[0.053685–0.068945]
Female (normal)	0.046941	0.042847	0.010993	0.015717	[0.038491–0.055392]
Female (abnormal)	0.057695	0.051591	0.018627	0.028651	[0.043377–0.072012]

The mean and median values showed more significant differences among women than men. Comparing the 95% confidence interval (CI) revealed that, without the influence of other factors, the overlapped intervals between aesthetic and unaesthetic were smaller among males than females. We set the lower bound of the 95% CI of unaesthetic average TDV for each gender as the cutoff value for analysis (Table 2B) to minimize errors that occur when the samples of males were aesthetic, and the samples of females were unaesthetic. The logistic regression model was not employed, but the cutoff value from the average TDV was directly obtained from the interpolation model. The error for aesthetic samples of men and unaesthetic samples of women was significantly decreased in the process, although the overall accuracy rate was also decreased.

## Discussion

We investigated the value of digitization of the 3D morphological features of the temporal region through the 3dMD Facial Stereo-photography System for subjective aesthetic evaluation and quantitative analysis of objective measurement data.

The study involved fitting a temporal morphology model by interpolation and evaluating the TDV as an indicator of aesthetic or unaesthetic features. The research combines quantitative mathematical analysis with subjective aesthetic evaluation to provide a generalized and objective evaluation method for temporal depression.

Temporal filling is a procedure used to correct temporal depression. Facial attractiveness is a major factor influencing personal image. The outcome of temporal filling can affect patient satisfaction and mental health (Liew et al., 2016).

The use of 3D-scanning devices to assess outcomes of surgery is becoming more popular, with 3dMD being one of the most currently used systems (Plooij et al., 2011; Weinberg et al., 2006). Postoperative evaluation of temporal augmentation typically relies on subjective judgment by physicians, which lacks an objective and unified standard.

The scanning model of 3dMD is considered to be precise, which is higher in its accuracy than using MRI on soft tissue surfaces, and its reliability and reproducibility for facial measurements have been proven. MRI (1.5T clinical MR Avanto scanner; Siemens Healthcare, Erlangen, Germany) is accurate to 1 mm (Varies with acquisition sequence and parameters such as slice thickness) while the 3dMDface system has its accuracy of 0.2 mm (Knoops et al., 2017).

Specific facial regions are sometimes defined based on 3D images (Donofrio et al., 2016; Jones et al., 2016). Previous studies have described the Vectra Software Suite® (VSS), which generates landmarks automatically to locate the temporal region (Camison et al., 2018; Hernandez et al., 2020). In this research, we defined the temporal area by touching the bony landmarks and marking the facial marker lines directly on the skin to avoid body surface projection deviations caused by shadows or image rendering. The marker lines were consistent with the bony landmarks, and we assessed the outcome of temporal augmentation (Cotofana et al., 2020), given reports of depression in the temporal region from clinical practice (Coleman & Grover, 2006).

Carruthers et al. (2016) conducted a large-sample trial involving subjective evaluation of temporal depression and designed a photo numeric scale. This method, which uses 2D photos to evaluate temporal depression, is user-friendly and fast.

Current practices for assessing temporal depression include blinded investigators using validated scales on two-dimensional photographs; there is a lack of a gold standard for temporal depression (Haidar et al., 2021). As we know, facial depression is subjective. Subjective evaluations were the main approaches used in the present study, but we quantified temporal depression through mathematical modeling combined with subjective and objective evaluations. Through 3dMD, the quantified data is free of errors caused by subjective observations and not influenced by structural shadows, skin color, ambient light, or other interferences. Furthermore, we achieved higher accuracy and reliability than 2D photos cannot achieve (Heike et al., 2009; Plooij et al., 2009). The study provides a threshold value of temporal depression through the mathematic model, which reflexes the subjective aesthetic criteria of the physicians. Calculating the TDV provides a specific parameter which could be used as a reference for plastic surgeons to adjust the filling volume and method.

In the rating process, the result retrieved indicated that most samples have unaesthetic temporal morphology. Aesthetically, the temporal morphology for most people is considered to be under the category of aesthetic; however, it does not equal to a morphologically perfect plane. In clinical practice, patients are dissatisfied with their appearance for various reasons, including psychological reasons, which contribute to overdemanding for the “perfect” temporal shape. In other words, even the slightest temporal morphological depression will be viewed as unaesthetic by patients themselves, and this scenario is what we mean by “aesthetically unaesthetic” but “morphological aesthetic”. The classification of unaesthetic includes some “morphological aesthetic” samples.

Most patients come to the clinic because of temporal depression; augmentation is often regarded as a phenomenon of obesity, and there is no need to have a temporal filling. In the research, augmentation is classified as “unaesthetic”, but temporal convexity is rare amongst patients of Asian origin due to the thicker subcutaneous layer; however, this means that the likelihood of age-related subcutaneous volume loss will be more pronounced (Liew et al., 2016; Shirakabe, Suzuki & Lam, 2003), and in our research, there is only one subject has augmentation.

The interpolation method that we used is often used to compute structural defects (Kodera, Nishitani & Okihara, 2020; Wang et al., 2018), correct and restore images (Qi, Yu & Xu, 2019), simulate human faces for identification (Xu & Wang, 2017), and reconstruct 3D leaf models (Oqielat, 2020). It has also been used to simulate shapes and analyze characteristics of eyebrows (Du et al., 2019). We applied this method in the field of facial aesthetics to generate temporal surfaces from specific points to analyze corresponding features.

The actual state of the temporal soft tissue was determined by evaluating the difference between the interpolated and actual surfaces. The interpolated surface was generated from characteristic points of the actual surface, and the relationships between these points can be regarded as features of the actual surface, which differ between individuals. The interpolated surface is a more continuous surface, which is considered to be more aesthetically pleasing on an actual human face.

The prediction accuracy rate of the research model was found to be 0.73 for men and 0.73 for women. In comparison to the accuracy rate of our mathematical model in terms of rating results, the model’s accuracy reaches the average accuracy rate of the first rating. Therefore, we hope this model could be developed as a pre-diagnostic tool. Before specialist evaluation, physicians could use this research model to make auxiliary diagnoses and improve the efficiency of diagnosis.

This study provides a quantitative analysis that transforms abstract aesthetic evaluations into digital differences, enabling the development of soft-tissue standards. The increased insight will enable more information to be provided to patients and physicians. More studies into soft tissues are required to finalize the development of the standards.

## Conclusions

This is the first study to transform qualitative morphological differences into quantitative numerical differences using 3D model trimming and mathematical analysis. This method has a high degree of overlap and good repeatability, indicating that subjective aesthetics have a low influence on the judgment of facial morphology. Our mathematical model is a useful tool for making auxiliary diagnoses relating to the contour of the temporal region.

## Supplemental Information

10.7717/peerj.14226/supp-1 Supplemental Information 1 The TDV of each model trimmed by the researchers and the final TDV calculated after removing the error data.

## Additional Information and Declarations

### Data Availability

The following information was supplied regarding data availability: The raw data is available as a Supplemental File.
